# Methods matter: Comparison of techniques used for sea anemone venom extraction

**DOI:** 10.1016/j.toxcx.2025.100219

**Published:** 2025-03-08

**Authors:** K.L. Kaposi, D.T. Wilson, A. Jones, J.E. Seymour

**Affiliations:** a College of Public Health, Medicine and Veterinary Science, James Cook University, Cairns, Queensland, 4878, Australia; b Australian Institute for Tropical Health and Medicine, James Cook University, Cairns, Queensland, 4878, Australia; c Institute for Molecular Bioscience, The University of Queensland, Brisbane, QLD, Australia

**Keywords:** Actiniaria, Cnidae, Toxins, Mass spectrometry, Proteomics

## Abstract

The study of cnidarian (coral, sea anemone, and jellyfish) venom provides important evolutionary and ecological insights and unlocks vast opportunities for biodiscovery of novel compounds. The success of the field is dependent on not only the acquisition of sufficient quantities of venom but also the ability to compare venom between species and studies. To date, no direct comparison of the main techniques used to acquire sea anemone venom has been performed to determine the comparability or validity of these methods to yield venom derived from within cnidarian venom apparatus – cnidae. This study aims to compare the venom extracted from a sea anemone via three common methods: isolated cnidae, electrostimulation, and physical manipulation. Using a range of non-targeted proteomic and mass spectrometric techniques, we showed each method yielded distinct differences in both the composition and abundance of components detected for extraction method. Furthermore, few identified components were shared between each of the extraction methods. These results highlight that different venom collection methods yield vastly different results. While further investigation is required, to validate the source of each of the components from within each sample, we argue that sample collection from isolated cnidae is likely to be the most representative of true venom components.

## Highlights

• Isolation of cnidae, electrostimulation, and physical manipulation are among common actiniarian venom collection methods.
• This study aimed to assess the comparability of these methods.
• Venom composition differed significantly across the three extraction methods.
• 1% of molecules were detected in venoms from all three extraction techniques.
• Venom from isolated cnidae are likely to most represent true actiniarian venom.

## Graphical abstract

**Figure:**
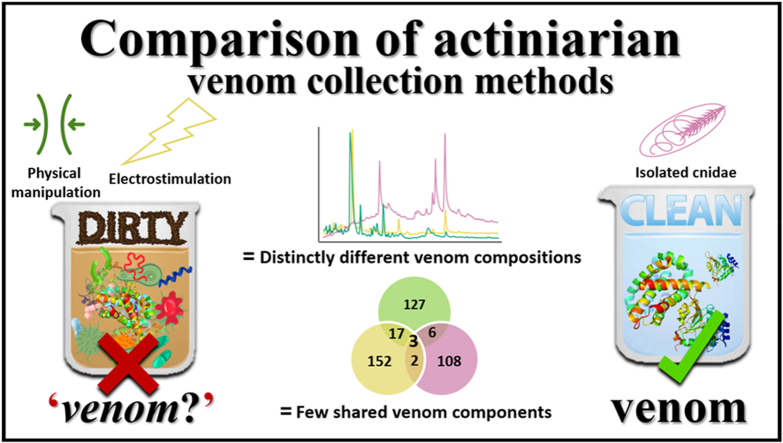

## 1 Introduction

Fifteen percent of the world's animals are venomous (Herzig, 2021), and are capable of injecting biological toxins into predators and prey directly through an inflicted wound (Casewell et al., 2013; Fry et al., 2009). Cnidarians (corals, sea anemones, and jellyfish) are among the earliest diverging lineage of venomous animals (Park et al., 2012) and possess a venom delivery system unique to their Phylum. Instead of a central venom gland and delivering venom through an apparatus, such as fangs or a stinger, cnidarian venom is scattered throughout their body in a multitude of specialized organelles, called cnidocysts (=cnidae) (Weill, 1929).

Broadly, cnidae are further classified into three groups; nematocysts, spirocysts, or ptychocysts (Mariscal, 1984). While spirocysts and ptychocysts are both described to be monotypic groups, there are approximately 30 different types of nematocysts reported (Mariscal, 1984). While it is widely reported that nematocysts are the sole group to contain venom, it is important to acknowledge that it is currently unknown whether all nematocysts contain venom (Mariscal, 1974, 1984), or whether venom may also be present within spirocysts. From this point forward, ‘cnidae’ and ‘nematocyst’ will be used interchangeably, and will also include reference to spirocysts, unless otherwise specified.

Fundamentally, nematocysts are miniature (3–250 μm) venom filled capsules, enclosing a tightly coiled tubule, (often armored with barbs and spines) and held under extreme pressure (Mariscal, 1974; Weill, 1929). Upon activation, the tubule within the nematocyst is ejected from within the capsule at extreme force, allowing them to penetrate the external tissue, whether it be skin, exoskeleton, or scales, of the target organisms (Holstein and Tardent, 1984; Nüchter et al., 2006; Weber, 1989), facilitating the injection of venom directly into the intended victim (Ewer, 1947; Hessinger and Lenhoff, 1973; Mariscal, 1974; Tardent, 1995). As such, nematocysts are often aptly described as highly sophisticated microscopic weapons (Tardent, 1995).

The ‘cnidarian venom’ held within nematocysts is a complex cocktail of toxic components composed of protein, peptides, and non-proteinaceous small molecules and should not be confused with poisonous toxins that may be secreted from, or contained within ectodermal gland cells of some species (Casewell et al., 2013; D’Ambra and Lauritano, 2020; Frazão et al., 2012; Fry et al., 2009; Jouiaei et al., 2015a,b; Moran et al., 2012; Zhang et al., 2003).

The study of venom not only provides important evolutionary and ecological insights into respective species, but also unlocks vast opportunities for biodiscovery of novel compounds that may be exploited for therapeutic or agrochemical products (Casewell et al., 2013; Fry et al., 2009; Herzig, 2021). The success of studying venoms, including those from cnidarians, is dependent on the ability to not only obtain sufficient quantities, but also being able to compare venom collected between species and studies.

The collection and study of cnidarian venom is challenging, largely due to the challenges of acquiring pure venom from the organism in sufficient quantities required for analyses (Cantoni et al., 2020). Historically, techniques used to acquire cnidarian venom can be summarized into five broad strategies: homogenization of whole tissues, electrostimulation, physical manipulation, chemical (ethanol, acetic acid, or potassium chloride) immersion, and rupture of isolated cnidae. With the exception of one study demonstrating that both the quality and bioactivity of *Chironex fleckeri* (box jellyfish) venom is impacted by method of collection (Cantoni et al., 2020), little consideration has been given towards how the venom extracted from other methods may differ (Frazão et al., 2012).

The study of cnidarian venoms is further complicated by the differences in the venom between functional areas of the body (Ashwood et al., 2021; Klompen et al., 2018, 2022; Little et al., 2020; Macrander et al., 2016; Mitchell et al., 2017; Prentis et al., 2018), intraspecific variation (Mitchell et al., 2020; Winter et al., 2010; Yue et al., 2019) and venom plasticity, which has been shown to occur within several species (Ben-Ari et al., 2018; Jouiaei et al., 2015a,b; O’Hara et al., 2018; Oakley et al., 2017; Richier et al., 2008; Sachkova et al., 2020; Sunagawa et al., 2009; Underwood and Seymour, 2007; Yosef et al., 2020).

Due to their medical importance, much attention has been devoted to optimizing venom collection methods from cubozoan jellyfish. For such species, the liberation of venom directly from nematocysts that have been isolated from the organism has emerged as the gold standard (Bloom et al., 1998; Cantoni et al., 2020; Carrette and Seymour, 2004). However, no such attention has been given to anthozoans, where any of the five broad venom collection methods have been used (Frazão et al., 2012). In addition to the shared challenge of requiring many thousands of cnidae, many of the techniques currently used to acquire anthozoan venom are associated with a high risk of contamination from mucus (Stabili et al., 2015), cellular debris (Mariottini and Pane, 2010), non-cnidocystic toxins (Moran et al., 2012; Zhang et al., 2003), and other organisms often associated with the animal (eg. zooxanthellae, bacteria, and viruses) (McCauley et al., 2023). This thus makes it difficult to determine which components of the extracts obtained by these methods are venom products derived from a nematocyst. Furthermore, without a thorough understanding of the limitations and variation among each of the methods, the ability to compare results between studies is impaired.

This study aimed to use a range of analytical techniques to compare the composition of venom extracted from a small sea anemone species, *Isactinia* sp., via three commonly used methods: isolated cnidae, electrostimulation, and physical manipulation. These methods were chosen as venom collected would theoretically only contain the venom originating from cnidae on external regions of the body (i.e. tentacles and body column), thereby reducing the sources of potential contamination and variability.

While venom components obtained from each method were identified and compared, the purpose of this study however was not to provide a complete, thorough catalogue of toxins that make up the venom arsenal within the *Isactina*-MTQ species.

## 2 Methods

### 2.1 Study organism

The tropical sea anemone, *Isactini*a-MTQ was selected as the model organism of this study. Type specimens may be referenced within the Museum of Tropical Queensland under the catalogue number ‘MTQ G80200’. This species typically ranges 0.5–5 cm in diameter (oral disc). Specimens ranging between 1 and 2 cm were haphazardly collected from an established population at James Cook University, Cairns (Australia). This sea anemone is known to engage in photosymbiosis with the zooxanthellae, *Cladocopium* (Kaposi et al., 2025), and has been identified to possess seven cnidae types (Kaposi et al., 2023). The cnidoms of external morphological features (tentacles and body column), consisted of basitrichs and spirocysts, whilst internally (actinopharynx and mesenterial filaments), cnidoms were made up of *b*-mastigophores, *p*-mastigophores, *p*-amastigophores, and basitrichs (Kaposi et al., 2023).

### 2.2 Venom collection methods

#### 2.2.1 Isolated cnidae

Cnidae were isolated from twenty sea anemones and ruptured using methods adapted from (Bloom et al., 1998; Carrette and Seymour, 2004) respectively. Sea anemones were anesthetised in 30 mL artificial seawater with a gradual titration of approximately 0.8 mL of 1 M magnesium chloride (MgCl_2_) over 30 min. Artificial seawater was prepared by mixing aquarium salt (Red Sea©, Riyadh, Saudi Arabia) with reverse-osmosis water to a concentration of 35‰ (±1‰, handheld refractometer). Individual narcoticised sea anemones were then transferred into 5 mL of fresh artificial sea water before being placed in −80 °C. After 1-h, frozen sea anemones were removed and left to thaw for 24 h at 4 °C. Thawing caused lysis of sea anemone tissue and after 24 h it was evident that the ectoderm of external tissues (tentacles and body column) had begun to slough from the rest of the body. A 3 mL plastic pasture pipette was used to gently aspirate and collect the lysed ectoderm and exudate. Preliminary investigations found that this method yielded a density ratio of 7.82 cnidae: 1 Symbiodiniaceae (unpublished data).

The collected ectoderm from each sea anemone was pooled together. Tip sonication at 70 Hz for 15 s was used to separate cnidae from ectodermal cells. The resulting homogenate was sieved through a 0.5 mm mesh, and then three times through a 70 μm cell strainer, removing larger particles and debris. The sample was then rinsed thrice in 30 mL artificial seawater via centrifugation for 5 min at 800 rcf (Rotina 420 R, Hettich©, Tuttlingen, Germany). All except 5 mL of supernatant was replaced with artificial seawater, and the pellet resuspended each time. After the final rinse, the pellet was transferred to a 1.5 mL Eppendorf tube and centrifuged for 1 min at 12,500 rcf (Mikro 185, Hettich©, Tuttlingen, Germany). The supernatant was discarded and replaced with 1 mL of reverse osmosis water, before sonication at 70 Hz for 5 s resuspended the pellet.

The sonicated solution was then transferred into micro-disruption tubes (SSIbio©, Lodi, United States) filled with approximately 1.2 g of 0.5 mm diameter glass beads (Cole-Parmer©, Vernon Hills, United States). Cnidae were disrupted using 15 x 2-min cycles on a mini bead beater (Biospec©, Bartlesville, United States) set at 5000 rpm, with a 2-min resting interval between each cycle. To maintain a constant temperature, and thus avoiding overheating and denaturing of the venom extract during cnidae disruption, bead beating was performed in a walk-in cold room (4 °C).

Once the disruption cycles were complete, samples were centrifuged for 1 min at 12,500 rcf, and supernatant collected. To ensure that the maximum amount of venom was collected, the glass beads were rinsed a further three times with an additional 1 mL reverse osmosis water, with vigorous shaking and subsequent centrifugation. This supernatant was collected and combined with the supernatant collected prior to the rinse. The resulting venom was frozen at −80 °C, and lyophilized.

#### 2.2.2 Electrostimulation

Adapted from the Malpezzi et al. (1993) protocol, five sea anemones were pooled in 10 mL of artificial seawater, and electrically stimulated by submerging two exposed copper wires with a current at 10 V for a period of 1 min (power source: Olympus Tokyo TE-II Optical Transformer Microscope Power Supply 220 V) into the surrounding water. To ensure that each sea anemone had even exposure time to the electrical current, the wires were rotated in the dish. Cnidae discharge was confirmed by inspecting each sea anemone under a stereomicroscope (Olympus SZ51) immediately after electrostimulation.

After electrostimulation, the resulting exudate was collected using a 3 mL plastic pasture pipette. An additional 5 mL of artificial seawater was used to rinse the sea anemones. This liquid was collected and pooled with the original exudate, prior to filtering through a 70 μm cell strainer thrice to remove debris. The exudate was then centrifuged for 5 min at 2000 rcf Mikro 185) and the resulting supernatant collected, frozen at −80 °C, and lyophilized.

#### 2.2.3 Physical manipulation

Following methods adapted from Senčič and Maček (1990) and Razpotnik et al. (2010), five sea anemones were subjected to physical manipulation. Sea anemones were placed into 5 mL of artificial seawater in the corner of a bisphenol-A and phthalate (BPA) free, food grade, plastic sandwich bag. Sea anemones were gently massaged by applying gentle pressure to the outside of the bag, whilst ensuring that the bag remained tilted. After 1 min of gentle physical manipulation, the resulting venom exudate was collected and filtered thrice through 70 μm cell strainer. The exudate was then centrifuged, frozen, and lyophilized.

### 2.3 Sample processing

Exudates obtained via the collection methods (isolated cnidae, electrostimulation, and squeezing methods) were transferred into an ultra-centrifugal filter (Millipore, Amicon®, Darmstadt, Germany) with a 3 kDa molecular weight cutoff. Microfiltration of samples were run at 4000 rcf for 40–90 min (Rotina 420 R). The <3 kDa permeate fraction and the > 3 kDa retentate fraction of each sample was collected. Collected fractions were lyophilized and stored at −80 °C until use.

Due to the low volumes and protein concentrations obtained, for each of the following analyses, fresh samples were obtained using the methods described above.

### 2.4 Sodium dodecyl sulfate polyacrylamide gel electrophoresis (SDS-PAGE)

The protein composition of exudates collected from each of the extraction methods were assessed by SDS-PAGE. The lyophilized >3 kDa retentate factions obtain from each venom collection method were resuspended using MilliQ water and the soluble protein content quantified using QuBit Protein Concentration Assay kit (‘QuBit assay’ hereafter). From each sample, 15 μg of soluble protein was prepared and combined with an equal volume Tricine Sample Buffer (Novex®, Waltham, United States) and denatured at 95 °C for 5 min. Samples were passed through a NuPAGE 10–20% Tris-Tricine gel at a rate of 90 V for 20 min and 120 V for 60 min with a constant amperage of 0.02 Å. Current was applied until the dye marker was within 1 cm of the gel bottom. Electrophoresis was performed with a Tricine SDS Running Buffer (Novex®) in a XCell SureLock™ mini electrophoresis cell (Thermo Fisher Scientific, Waltham, United States). Spectra Multicolour Low Range Protein Ladder (Thermo Fisher Scientific) was employed on either side of the venom samples as a molecular weight (1.7–40 kDa) marker. Protein bands were visualised using Imperial Protein Stain (Thermo Fisher Scientific). The densitometric analysis and peak profiles were plotted for the gel images using GelAnalyzer (v23.1.1).

### 2.5 Liquid chromatography/electrospray ionisation mass spectrometry (LC/ESI-MS)

Liquid chromatography was used to assess the composition of soluble peptides and proteins within the <3 kDa permeate and >3 kDa retentate fractions for each method. The lyophilized samples were reconstituted using MilliQ water and the soluble protein content quantified using a QuBit assay. Equal quantities of soluble protein from each of the <3 kDa permeate (100 μg protein) and >3 kDa retentate (60 μg protein) fractions were prepared and made up to a volume of 75 μL and 48 μL with MilliQ respectively. Equal volumes of LCMS solvent A (0.1% formic acid) was added to each sample.

Samples were analysed using a Shimadzu® (Kyoto, Japan) LC/ESI-MS-2020 mass spectrometer with a Phenomenex 3.6 μm PEPTIDE XB-C18 (100 Å, 150 × 2.21 mm) LC column (Phenomenex®, Torrance, United States) at 30 °C. Sample of < 3 kDa permeate (90 μL) and > 3 kDa retentate (100 μL), was loaded onto the LC/ESI-MS via an autosampler (Shimadzu SIL-20AC_HT_) at a flow rate of 250 μL/min and the absorbance monitored at 214 nm and 280 nm. Sample elution included a desalting step using 100% of solvent A (0.1% formic acid) for 10 min, followed by a 1% gradient of solvent B (90% acetonitrile, 0.1% formic acid); 0–80% for 80 min, 80–90% for 5 min, 90% for 5 min. Mass spectra were collected in positive ion mode (scan range; m/z 160–2000), and negative ion mode (scan range; m/z 200–2000), using a detector voltage of 1.30 kV, nebulizing gas flow of 1.5 L/min and drying gas flow of 3.0 L/min. Data was collected and analysed using LabSolutions software (version 5.96). Chromatograms were normalised by relative intensity, visualised using the ‘ggplot2’ package (Wickham, 2016) in R (version 4.0.1) and visually compared.

### 2.6 High resolution ultra-high-performance liquid chromatography, mass spectrometry (HPLC-MS)

A top-down proteomic approach was taken to determine and compare the composition of exudates from each of the venom collection methods. Similarly, equal quantities of soluble protein from each of the <3 kDa permeate (89 μg protein) and >3 kDa retentate (126 μg protein) fractions were prepared for mass spectrometry.

Mass spectrometry was performed at the University of Queensland’s Institute for Molecular Bioscience (IMB). Venom extracts were redissolved in 30% acetonitrile, 70% 0.1% formic acid in water. The extracts were analysed by uHPLC-MS on a Shimadzu Nexera uHPLC, LC30 coupled to a Triple time-of-flight (ToF) 5600 mass spectrometer (AB SCIEX, Framingham, United States) equipped with a duo electrospray ion source. Each extract (10 μL) was injected onto a Gemini C4 3 μm, 2 mm × 150 mm column (Phenomenex®) at 300 μL/min. Linear gradients of 2% solvent B for 0.5 min, 2–80% solvent B over 50 min, followed by 80–98% over 2 min were used and solvent B was held at 98% for 3 min for washing the column and returned to 2% solvent B for equilibration prior to the next sample injection. Solvent A consisted of 0.1% formic acid (aq) and solvent B contained acetonitrile/0.1% formic acid (aq). The ion spray voltage was set to 5500 V, declustering potential (DP) 100 V, curtain gas flow 25, nebuliser gas 1 (GS1) 50, GS2 to 60, interface heater at 150 °C and the turbo heater to 500 °C. The mass spectrometer acquired 500ms full scan ToF-MS data from m/z 400–2200 for mass profile analysis. The data was acquired and processed using Analyst TF 1.6 software (AB SCIEX, Canada). PeakView® 2.1 (AB Sciex Pte. Ltd., Singapore) software was used to identify and analyse the resulting spectra.

The total ion current (TIC) for each exudate was visualised using the ‘ggplot2’ package (Wickham, 2016). in R (version 4.0.1).

The ion series was automatically reconstructed using the PeakView® BioTools module and filtered for ions above the intensity threshold of 1000 counts per second (cps). The spectrum of each of the remaining ions were inspected and individually validated. Ions were considered to be shared between samples if they had a mass difference less than 0.5 Da and eluted within 0.2 min of each other. In cases whereby their mass was deemed to be the same (±0.5 Da), but the difference in their elution time was 0.2–3.5 min, these components were tentatively identified as being the same.

The number of confirmed ions (venom components) shared within the exudates for each of the venom collection methods was quantified and visualised in R with an Upset plot using the ‘UpSetR’ package (Conway et al., 2017), whereby the sets were the venom collection methods, and the intersections were the venom components found to have been common between the methods. Jaccard similarity was used to further quantify the similarity of venom components from within each of the exudates among one another. Tentatively shared components were kept separate for these analyses.

## 3 Results

### 3.1 SDS-PAGE

**Fig. 1:**
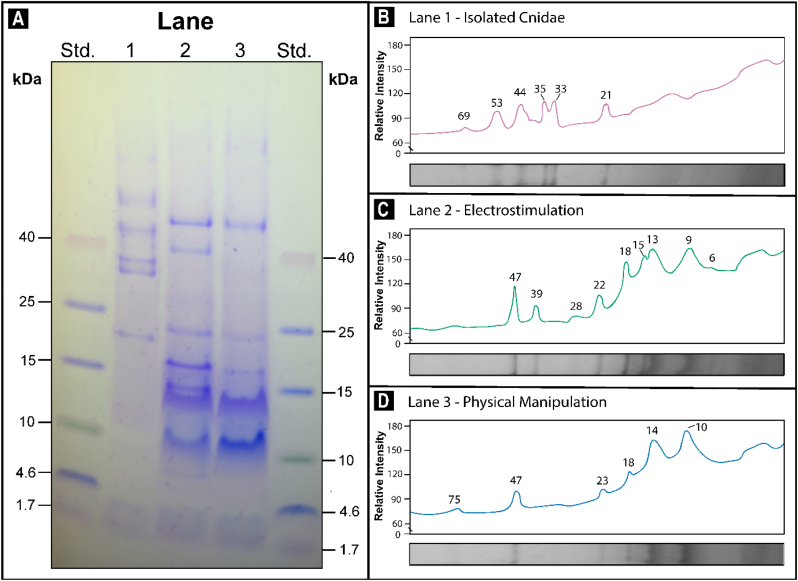
**a)** Comparative electrophoretic profiles of the soluble protein composition of the >3 kDa retentate fractions of exudates collected from the sea anemone *Isactinia*-MTQ using three venom extraction methods: isolated cnidae (Lane 1), electrostimulation (Lane 2), and physical manipulation (Lane 3). The molecular weight (kDa) of protein standards (Std) are shown. Peak profile obtained by densitometric analysis for exudates from the **b)** isolated cnidae (magenta), **c)** electrostimulation (teal), **d)** physical manipulation (blue) venom collection methods. The estimated molecular weights (kDa) of protein bands expressed are labelled. (For interpretation of the references to colour in this figure legend, the reader is referred to the Web version of this article.)

SDS-PAGE revealed distinct differences in the both the molecular weights and intensity of the soluble proteome detected within each of the exudates obtained from the physical manipulated, electrostimulated and isolated cnidae venom extractions from *Isactinia*-MTQ (Fig. 1a). Electrophoretic profiles of exudates recovered from isolated cnidae revealed six distinct protein bands (Fig. 1b). Evidence of these same protein bands was absent from the venoms recovered via electrostimulation and physical manipulation (Fig. 1c and d). Exudates acquired through electrostimulation displayed the greatest complexity in protein composition, with nine prominent protein bands revealed (Fig. 1c). In comparison, exudates extracted through physical manipulation displayed only six protein bands (Fig. 1d). Exudates obtained through physical manipulation displayed the widest distribution of soluble protein from the exudates tested, with bands ranging from 10 to 75 kDa (Fig. 1d). Compared to exudates from isolated cnidae and electrostimulation, which yielded protein size distributions of 21–69 kDa (Fig. 1b) and 6–47 kDa (Fig. 1d), respectively. Two proteins, 18 and 47 kDa, were shared between the exudates acquired through electrostimulation and physical manipulation, both of which appeared at a greater intensity in the electrostimulation extract.

### 3.2 LC/ESI-MS

**Fig. 2:**
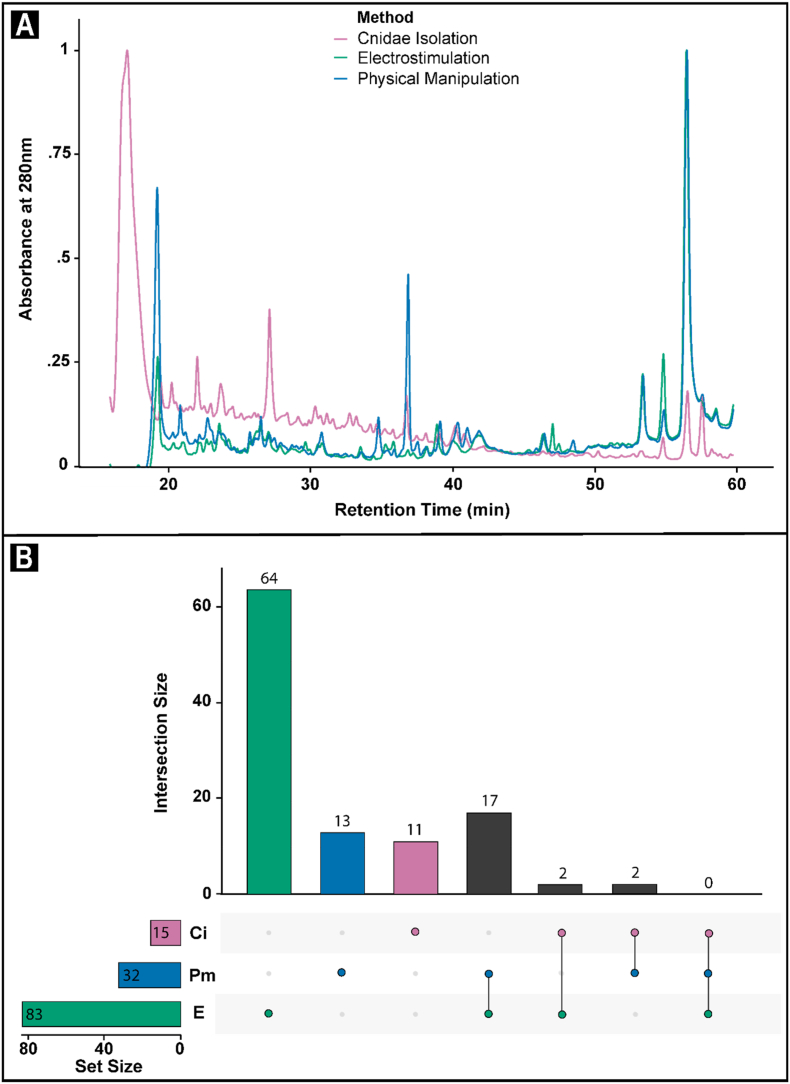
**(a)** Normalised absorbance at 280 nm between 16 and 60 min in chromatograms for the <3 kDa permeate fraction of venom exudates collected from the sea anemone *Isactinia*-MTQ using the isolated cnidae (magenta), electrostimulation (teal), and physical manipulation (blue) methods. **(b)** Upset plots illustrating the number of unique and shared molecules identified through multi-charged ion analysis in each of the isolated cnidae (Ci, magenta), electrostimulation (E, teal), and physical manipulation (Pm, blue) methods. (For interpretation of the references to colour in this figure legend, the reader is referred to the Web version of this article.)

**Fig. 3:**
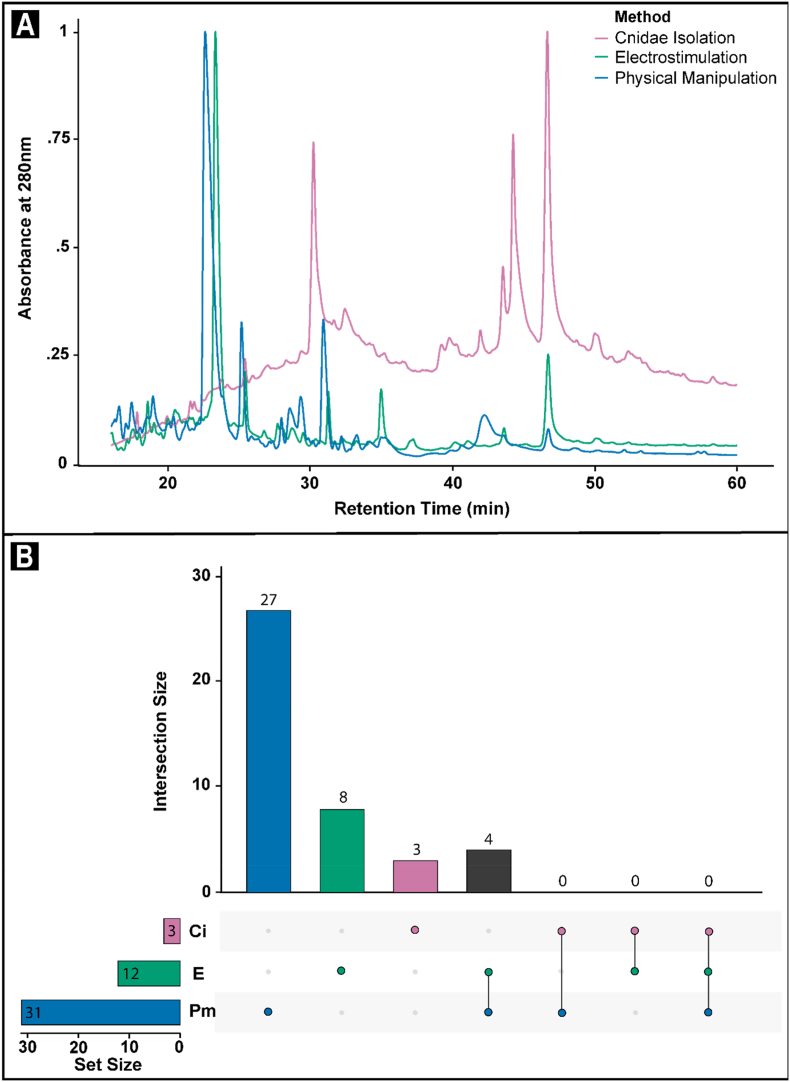
**(a)** Normalised absorbance at 280 nm between 16 and 60 min in chromatograms for the >3 kDa retenate fraction of venom exudates collected from the sea anemone *Isactinia*-MTQ using the isolated cnidae (magenta), electrostimulation (teal), and physical manipulation (blue) methods. **(b)** Upset plots illustrating the number of unique and shared molecules identified through multi-charged ion analysis in each of the isolated cnidae (Ci, magenta), electrostimulation (E, teal), and physical manipulation (Pm, blue) methods. (For interpretation of the references to colour in this figure legend, the reader is referred to the Web version of this article.)

The intensity and complexity of chromatograms of the 280 nm wavelength chromatograms differed significantly between collection methods in both the <3 kDa permeate and >3 kDa retentate fractions (Fig. 2, Fig. 3a). In the <3 kDa permeate fractions, exudates from isolated cnidae show the presence of more intense peaks than both the exudates collected via electrostimulation and physical manipulation (Fig. 2a). The 280 nm chromatogram for the <3 kDa permeate fractions of electrostimulation and physical manipulation exudates were similar, both in presence and intensity of peaks (Fig. 2a).

Multi-charged ion analysis identified and confirmed the presence of 15 molecules from with the ioslated cnidae exudates, 83 from the electrostimulation exudates, and 32 from physically manipulated exudates (Fig. 2b; Supplementary Table 1). These molecules ranged from 0.17 to 0.52 kDa, 0.21–0.56 kDa, and 0.21–1.84 kDa, respectively (Supplementary Table 1). No molecules were shared within all three exudates (Fig. 2b; Supplementary Table 1). Two molecules were shared between the cnidae and each of the other exudates (Fig. 2b). Seventeen molecules were shared between the electrostimulation and physical manipulation samples (Fig. 2b). The remaining molecules were exclusive to their respective sample (Fig. 2b). Based on the presence/absence data of 109 identified venom components, the Jaccard Similarity Index between isolated cnidae and electrostimulation, and isolated cnidae and physical manipulation exudates was 0.021 and 0.044 respectively. Conversely, the Jaccard Similarity Index between electrostimulation, and physical manipulation exudates was 0.17. Together, these values indicate that while exudates from electrostimulation and physical manipulation were 17% similar, they each had less than 5% similarly with exudate from isolated cnidae.

In the >3 kDa retentate fractions, the 280 nm chromatograms were more variable (Fig. 3a). The most intense peaks occurred in the exudates collected using physical manipulation (Fig. 3a). Multi-charged ion analysis identified and confirmed the presence of 3 molecules within the isolated cnidae exudates, 12 from electrostimulation exudates, and 31 from the physically manipulated exudates (Fig. 3b–Supplementary Table 2). These molecules ranged from 0.23 to 16.25 kDa, 0.16–14.61 kDa, and 1.89–9.16 kDa, respectively (Supplementary Table 2). Whilst four molecules were shared between the electrostimulation and physical manipulation exudates, the remaining molecules were exclusive to their respective sample (Fig. 3b). Based on the presence/absence data of the 42 identified venom components, the Jaccard Similarity Index between electrostimulation, and physical manipulation exudates was 0.10. Conversely, the Jaccard Similarity Index between cnidae isolation with both electrostimulation, and physical manipulation exudates was 0.00. Together, these values indicate that while exudates from electrostimulation and physical manipulation were 10% similar, there was no similarly with exudate from isolated cnidae.

### 3.3 High resolution HPLC-MS

**Fig. 4:**
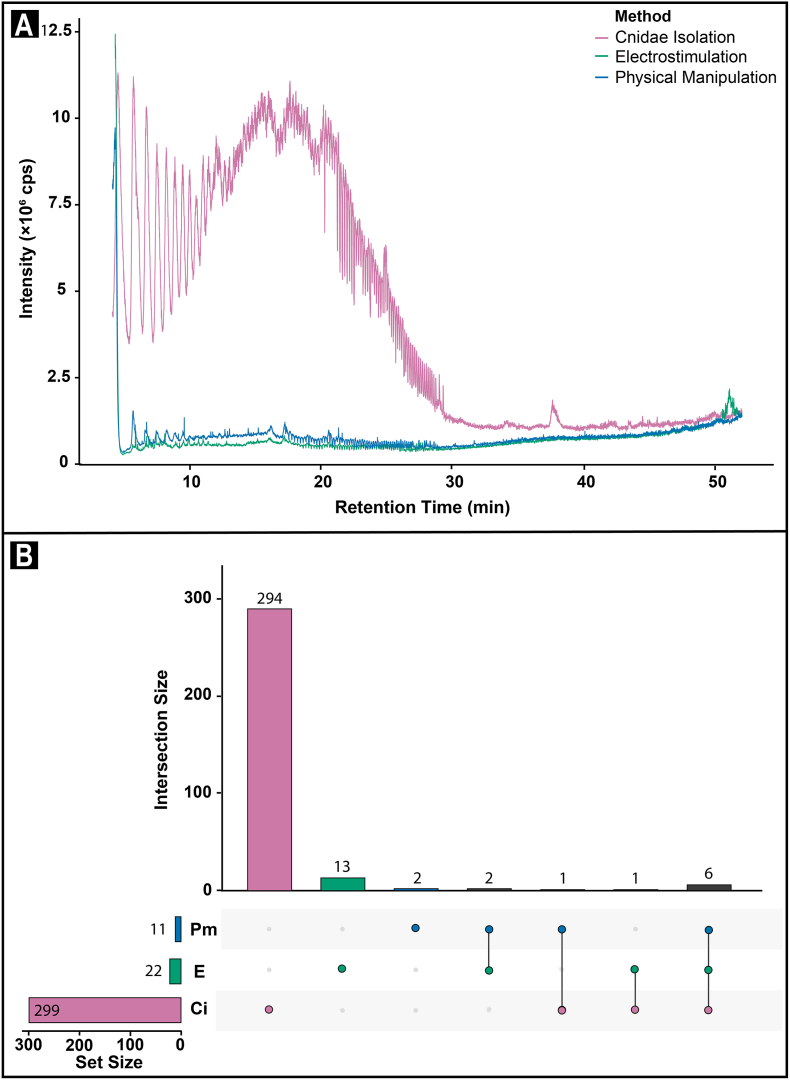
**(a)** Comparative intensity (counts per second) of the total ion current (TIC) chromatograms of <3 kDa permeate fraction of venom collected from sea anemone Isactinia-MTQ, using the isolated cnidae (magenta), electrostimulation (teal), and physical manipulation (blue) methods. **(b)** Upset plots illustrating the number of unique and shared molecules identified through uHPLC-MS in each of the isolated cnidae (Ci, magenta), electrostimulation (E, teal), and physical manipulation (Pm, blue) methods. (For interpretation of the references to colour in this figure legend, the reader is referred to the Web version of this article.)

A total of 299, 22, and 11 molecules were detected and manually confirmed with the ion intensity cutoff filter set to 1000 cps for the <3 kDa permeate fractions from the isolated cnidae, electrostimulation, and physical manipulation, exudates respectively (Fig. 4a, Supplementary Table 3). The molecules each ranged from 0.4 to 4.8 kDa in size. Only six of these molecules were shared between all three exudates (Fig. 4b). One molecule was shared between the cnidae and physical manipulation samples, two between the electrostimulation and physical manipulation samples, and one between cnidae and electrostimulation (Fig. 4b). Based on the presence/absence data of 316 identified venom components, the Jaccard Similarity Index between isolated cnidae and electrostimulation, and isolated cnidae and physical manipulation exudates was 0.022 and 0.023 respectively. Conversely, the Jaccard Similarity Index between electrostimulation, and physical manipulation exudates was 0.32. Together, these values indicate that while exudates from electrostimulation and physical manipulation were 32% similar, they each only had 2% similarity with exudate from isolated cnidae.

**Fig. 5:**
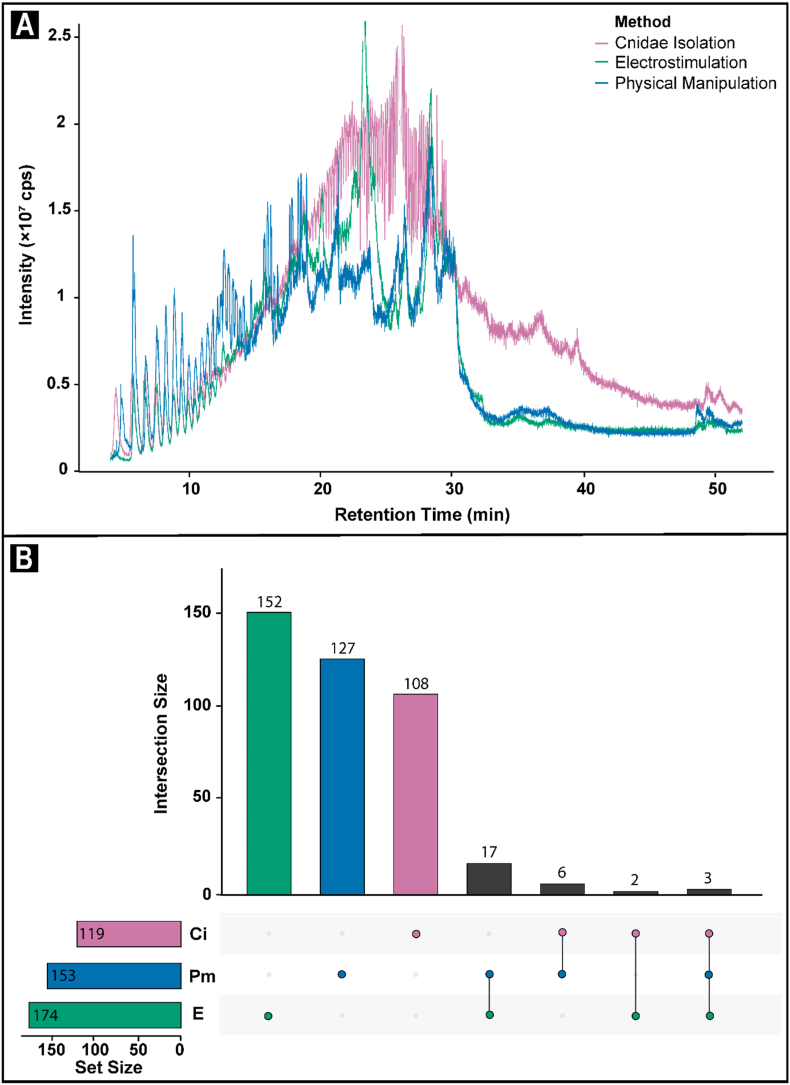
**(a)** Comparative intensity (counts per second) of the total ion current (TIC) chromatograms of >3 kDa retentate fraction of venom collected from sea anemone *Isactinia*-MTQ, using the isolated cnidae (magenta), electrostimulation (teal), and physical manipulation (blue) methods. **(b)** Upset plots illustrating the number of unique and shared molecules identified through uHPLC-MS in each of the isolated cnidae (Ci, magenta), electrostimulation (E, teal), and physical manipulation (Pm, blue) methods. (For interpretation of the references to colour in this figure legend, the reader is referred to the Web version of this article.)

A total of 119, 174, and 153 molecules were detected and manually confirmed with the ion intensity cutoff filter set to 1000 cps for the >3 kDa retentate fractions from the isolated cnidae, electrostimulation, and physical manipulation, exudates respectively (Fig. 5a, Supplementary Table 4). The molecules each ranged from 0.4 to 14.7 kDa in size. Only six of these molecules were shared between all three exudates (Fig. 5b). Three of these molecules were shared between all three exudates, six were shared amongst the cnidae and physical manipulation samples, 17 between the electrostimulation and physical manipulation samples, and two between cnidae and electrostimulation (Fig. 5b). Based on the presence/absence data of 415 identified venom components, the Jaccard Similarity Index between isolated cnidae and electrostimulation, and isolated cnidae and physical manipulation exudates was 0.017 and 0.034 respectively. Conversely, the Jaccard Similarity Index between electrostimulation, and physical manipulation exudates was 0.065. These values indicate that all samples shared less than 7% similarity.

## 4 Discussion

Using a range of non-targeted proteomic and mass spectrometric techniques, this study sought to quantify the comparability of three techniques (isolated cnidae, electrostimulation, and physical manipulation) commonly used to extract venom from sea anemones. Comparisons of the electrophoretic profiles, 280 nm wavelength and total ion current chromatograms, and ion masses revealed that the venom components detected within each of the exudates significantly differed between venom extraction methods. While samples obtained via electrostimulation and physical manipulation were determined to be up to 17% similar, these samples had less than 5% similarity with isolated cnidae extracts. Notably, only 1% of venom components were shared across all methods, raising questions as to the degree of true venom able to be detected within each of the extracts. Furthermore, as the majority of molecules detected within the electrostimulation and physical manipulation exudates were absent within exudates obtained from isolated cnidae, it is probable many of these are contaminants. The results of this study ultimately highlight the significant role that the choice of method in which the venom is collected has on the results obtained. Given that sea anemone venom is fundamentally, by definition, derived from within their specialized venom delivery system - cnidae, this study demonstrates the importance for future actiniarian venomic studies to strive to target venom from isolated cnidae, thereby reducing likely sources contamination likely present in other methods.

Across all proteomic and mass spectrometric analyses, over 700 components, ranging from 0.4 to 69 kDa, were identified from excudates obtained from the cnidae isolated from the sea anemone *Isactinia*-MTQ. A similarly high abundance of components (n = 499) has also been reported in venom extracted from cnidae isolated from the jellyfish, *Nemopilema nomurai* (syn. *Stomolophus meleagris*) (Li et al., 2014). In contrast, venoms from cnidae obtained from jellyfish *Cyanea nozakii, Chryssaora caliparea* and *Lychnorhiza malayensis* and sea anemone *Nematostella vectensis* were each reported to have significantly fewer components (n = 20–40) (Moran et al., 2013; Riyas et al., 2021), suggesting that the complexity of venom may differ between species. Furthermore, evidence also suggests that not all cnidae-derived venom components are toxins (Li et al., 2014; Riyas et al., 2021). In some jellyfish species, it is estimated that as much as 75% of all venom components may be associated with biological process, cellular structure, and molecular function (Li et al., 2014; Riyas et al., 2021).

Sea anemone venom can cause a range of physiological responses, including nerve damage, muscle inflammation, cell lysis and acute pain (Madio et al., 2019). The size of venom components identified within the exudates obtained from isolated cnidae fall within the sizes previously reported to be present within sea anemone venom. It is well documented that sea anemone venom typically ranges from 3 kDa up to 200 (Frazão et al., 2012; Honma and Shiomi, 2006; Madio et al., 2017) components within the 3–6.5 kDa are typically classed as neurotoxins, acting on voltage-gated ion channels (Castañeda et al., 1995; Cotton et al., 1997; Honma and Shiomi, 2006; Lehnert et al., 2014; Norton, 1991, 2009; Schweitz et al., 1995). Components within 18–20 kDa are generally pore-forming cytolysins (=actinoporins) (Anderluh and Maček, 2002; Norton, 2009; Turk, 1991). Whilst many components within the 13–45 kDa range have been reported to include enzymes such as phospholipase A2, which have been shown to have hemolytic and cytotoxic activities (Jouiaei et al., 2015a,b; Madio et al., 2019; Nevalainen et al., 2004).

It remains unknown what the function of each of the components identified from *Isactinia*-MTQ may be. Within a species, venom compositions may vary between distinct nematocysts types (McClounan and Seymour, 2012), corresponding to the specific ecological function of the respective cnidae (Shick, 1991). Similarly to (syn. *S. meleagris* (Calder, 1983), exudates obtained from *Isactinia*-MTQ were also composed of four distinct cnidae types (Kaposi et al., 2023). We hypothosise that the cnidae exudates obtained from *Isactinia*-MTQ would likely contain a similar proportion of toxin to non-toxin components as reported for *S. meleagris* (Li et al., 2014). The implementation of higher resolution mass spectrometry and transcriptomics would be required to potentially distinguish between the putative venom toxins present from any non-venomous components. Analysis of the artificial seawater, subjected to the same extraction methods, would also be beneficial in future studies to further identify potential contamination sources.

While the micro-extraction of venom from single cnidae organelles would ultimately achieve the most definitive results, of all techniques currently available, isolating cnidae is likely to result in the best representation of sea anemone venom. The ability for this method to successfully obtain biologically active venom has been validated in box jellyfish (Ramasamy et al., 2005). Through a direct comparison of the protein composition and toxicity of exudates obtained from isolated cnidae and the remaining cnidae-free tentacular tissue, it was confirmed that cnidae, and not other cellular tissues, was indeed the source of venom (Ramasamy et al., 2005).

The downside to isolated cnidae is that it is time-consuming and labor-intensive, requiring access to specialized equipment, isolation significantly reduces the risk of source contamination from exogenous, non-cnidae derived proteins, such as those related to other cells, symbionts, mucus and poisonous toxins that may be secreted from gland cells (Madio et al., 2017). The inevitable presence of small volumes of such contamination are unlikely to be detected in any considerable levels (Fu et al., 2021). As they will likely be masked by the relatively high concentration of components liberated from the cnidae, which has been shown to be positively correlated to the percentage of discharged nematocysts (Carrette and Seymour, 2004).

The use of electrostimulation is often regarded as one of the most effective methods for inducing nematocyst discharge and venom extraction from sea anemones (Frazão et al., 2012; Fu et al., 2021; Madio et al., 2017; Rifkin, 1982). Low risk of contamination and high survivability of sea anemones are touted as some of the main advantages of this method (Frazão et al., 2012; Fu et al., 2021; Madio et al., 2017; Rifkin, 1982). However, electrostimulation is known to also trigger electroporation and leakage of cellular contents (Movahed and Li, 2011; Tsong, 1991). Indeed, electroporation and the subsequent rupture and lysis of cells is among the preferred strategies commonly used to specifically extract cellular proteins (Haberl Meglic et al., 2015; Movahed and Li, 2011).

In contrast to the exudates obtained from the isolated cnidae, only 170 venom components (0.4–47 kDa) were identified in the exudates acquired through the electrostimulation of sea anemones. Moreover, few of these components were also detected from exudates of isolated cnidae, raising questions as to their source. While the presence of cnidae-derived venom components within the electrically stimulated exudates cannot be ruled out (Fu et al., 2021), it is clear that this method resulted in many components not seen in the cnidae samples. The presence of salt within biological samples also has the capacity to hinder analytical tests (Metwally et al., 2015; Piwowar et al., 2009). As ultra-centrifugal filters are commonly used to desalt biological samples (Amicron®, 2014), it is also possible salt, particularly in the <3 kDa permeate fractions, interfered with the detection of some low abundance venom components (Metwally et al., 2015; Piwowar et al., 2009).

Similarly to electrostimulation, the technique of applying pressure and physically manipulating the sea anemones is among one of the most common methods used to extract actinarian venom. While relatively cheap, quick and easy to perform, this method is also praised as being less harmful to sea anemones whilst reportedly allowing for the collection of pure venom (Fu et al., 2021; Hoepner et al., 2019; Senčič and Maček, 1990).

Exudates obtained via physical manipulation of *Isactinia*-MTQ resulted in the identification of approximately 150 venom components (0.4–75 kDa), most of which were not seen in exudates acquired using other methods. Due to the very low number of components shared, particularly from the isolated cnidae, it is difficult to ascertain whether these components originated in the venom yielding cnidae. Exudates retrieved through the physical manipulation of sea anemones are typically in the form of secreted mucus and surrounding sea water (Fu et al., 2021; Sintsova et al., 2018).

In its aqueous state, sea anemone mucus is approximately 96% water (Stabili et al., 2015). Once dehydrated, proteins were found to make up only 24% of the dry mucus weight from *Actinia equina,* whereby the remaining residuals were identified as predominately inorganic salts and carbohydrates (Stabili et al., 2015). Further to this, most of the proteins from within *Bunodosoma caissarum* mucus have been identified as putative toxins (Mazzi Esquinca et al., 2023). Sea anemone mucus have also been shown to have toxinological effects (Hoepner et al., 2019; Sintsova et al., 2018; Stabili et al., 2015) While the toxins within actiniarian mucus are biologically relevant, whether these toxins are from ‘venom’, capable of being injected into opposing organisms, remains contensious. Many non-venomous proteins, recruited from non-cnidae sources, may display similar traits and be easily misidentified as venom toxins (Ashwood et al., 2021; Fry et al., 2009). It is thus possible these toxins may be secreted from ectodermal gland cells (Moran et al., 2012; Zhang et al., 2003), or be just general housekeeping proteins from the mucus itself (Ramírez-Carreto et al., 2019; Stabili et al., 2015). Further investigation is required before adequate predictions can be made as to whether or not true venom is present, albeit perhaps in low abundance, within the products produced through this method.

## 5 Conclusion

Using the small sea anemone, *Isactinia-*MTQ, this study sought to elucidate the differences of three techniques (isolated cnidae, electrostimulation, and physical manipulation), commonly used to acquire actiniarian venom. A series of analyses highlighted significant differences and low similarities in the composition of the exudates obtained through each of these methods. As sea anemone venom is fundamentally derived from within cnidae, the low abundance of venom components shared between the isolated cnidae, electrically stimulated and physically manipulated exudates suggests that these latter methods are unlikely to yield true venom. Further testing on other species, along with the implementation of molecular biological techniques, such as transcriptomics, is recommended to validate this application of isolating cnidae to acquire true venom in other anthozoan species. Nonetheless, we recommend future studies wishing to comprehensively examine sea anemone venom should consider making all efforts to mitigate the risk of exogenous contaminants of non-cnidocystic origin. To this end, the results presented here suggest that this may best be achieved by liberating the contents of isolated cnidae.

## CRediT authorship contribution statement

**K.L. Kaposi:** Writing – original draft, Methodology, Investigation, Formal analysis, Data curation, Conceptualization. **D.T. Wilson:** Writing – review & editing, Supervision, Methodology, Formal analysis, Conceptualization. **A. Jones:** Writing – review & editing, Resources, Methodology, Formal analysis. **J.E. Seymour:** Writing – review & editing, Supervision, Resources, Conceptualization.

## Declaration of competing interest

The authors declare that they have no known competing financial interests or personal relationships that could have appeared to influence the work reported in this paper.

## Data Availability

Data will be made available on request.
