# Supplementary material for: Methods matter: Comparison of techniques used for sea anemone venom extraction
Source: Toxicon X. 2025 Mar 8;26:100219. doi: 10.1016/j.toxcx.2025.100219 (PMC11954122; doi:10.1016/j.toxcx.2025.100219)
Supplement: Supplementary Table 1 [file mmc1.doc]

**Methods Matter: Comparison of techniques used for sea anemone venom extraction**

Authors: Kaposi K.L^1^,^2^*, Wilson, D.T^2^, Jones, A.^3^ and Seymour J.E^2^

**Supplementary Table 1:** Monoisotopic mass (Da) and retention time (min) of molecules identified from within peaks of interest seen within the 280 nm chromatogram for the <3 kDa venom fractions collected from sea anemone *Isactinia*-MTQ, using the isolated cnidae (Cn), electrostimulation (E), and physical manipulation (Pm) methods.

| **Retention Time (min)** | **Monoisotopic Mass (Da)** | | |
| --- | --- | --- | --- |
|  | **Cn** | **E** | **Pm** |
| 12.8 | 171.931 |  |  |
| 12.8 | 307.895 |  |  |
| 16.8 | 203.99 |  |  |
| 16.8 | 515.29 |  |  |
| 19.5 |  |  | 471.818 |
| 19.5 |  |  | 1836.41 |
| 20.6 | 260.031 |  |  |
| 20.8 | 301.092 |  |  |
| 21 |  |  | 523.272 |
| 21.09 | 181.978 |  |  |
| 23.3 |  |  | 238.055 |
| 23.3–23.4 |  | 255.076 | 255.046 |
| 23.3–23.4 |  | 260.012 | 260.034 |
| 23.46 | 294.016 |  |  |
| 25.7 |  |  | 282.053 |
| 25.7 | 299.092 |  | 299.098 |
| 25.7 |  |  | 303.663 |
| 25.9 |  | 299.11 |  |
| 27.1 |  | 221.034 |  |
| 27.1 |  | 223.003 |  |
| 27.1 |  | 225.972 |  |
| 27.5–27.6 | 333.026 |  | 333.132 |
| 27.5–27.7 | 343.138 | 343.098 |  |
| 28.7 |  | 339.726 | 339.155 |
| 29.3 |  | 387.167 |  |
| 29.9 |  | 265.068 |  |
| 29.9 |  | 269.988 |  |
| 30.4 |  | 206.037 |  |
| 30.7–30.8 |  | 431.189 | 431.155 |
| 32.0–32.4 | 475.259 | 475.283 |  |
| 32.4 |  | 250.067 | 250.075 |
| 32.4 |  | 272.035 | 272.055 |
| 32.4 |  | 309.036 |  |
| 32.4 |  | 314.007 |  |
| 33.3–33.4 |  | 452.282 | 452.302 |
| 33.3–33.4 |  | 474.275 | 474.283 |
| 34.2–34.3 |  | 311.26 | 311.131 |
| 34.6 |  | 563.364 |  |
| 35.4 |  | 294.099 |  |
| 35.4 |  | 311.102 | 311.083 |
| 35.4 |  | 316.066 | 316.05 |
| 35.5 |  | 294.086 |  |
| 35.5 |  | 316.523 |  |
| 35.5 |  | 353.092 |  |
| 35.7 |  | 355.151 |  |
| 36.9 |  | 355.144 | 355.103 |
| 38.2 |  | 399.171 |  |
| 39.2–39.3 |  | 333.995 | 334.03 |
| 39.7 |  | 268.055 |  |
| 40.2 |  | 246.037 |  |
| 40.2 |  | 263.075 |  |
| 40.2 |  | 268.938 |  |
| 41.8 |  | 260.073 |  |
| 41.8 |  | 277.073 |  |
| 41.8 |  | 282.02 |  |
| 42.5 |  | 319.028 |  |
| 42.5 |  | 324.017 |  |
| 42.9 |  | 321.099 |  |
| 43.1 |  | 326.07 |  |
| 43.9 |  | 348.094 |  |
| 43.9 |  | 365.139 |  |
| 43.9 |  | 370.096 |  |
| 44.4 |  | 260.065 |  |
| 44.4 |  | 365.133 |  |
| 44.9 |  | 326.038 |  |
| 44.9 |  | 326.05 |  |
| 44.9 |  | 409.193 |  |
| 45.7 |  | 365.146 |  |
| 45.7 |  | 370.101 |  |
| 45.9 |  | 260.046 |  |
| 45.9 |  | 277.077 |  |
| 46 |  | 321.101 |  |
| 46.4 |  | 409.194 |  |
| 46.7 |  | 365.146 |  |
| 46.7 |  | 370.099 |  |
| 46.9 |  |  | 226.085 |
| 46.9 | 385.195 |  |  |
| 47.3 |  | 321.12 |  |
| 47.3 |  | 326.072 |  |
| 47.3 |  | 365.141 |  |
| 47.9 |  | 409.203 |  |
| 48.6 |  | 365.146 |  |
| 48.6 |  | 453.252 |  |
| 49.6 |  | 409.201 |  |
| 51 |  |  | 243.106 |
| 51.4 |  | 393.156 |  |
| 51.4 |  | 398.123 |  |
| 57.6 |  | 208.007 | 208.006 |
| 57.6 |  | 226.142 |  |
| 57.6 |  | 249.066 |  |
| 57.6 |  | 273.135 |  |
| 57.6 |  | 317.066 |  |
| 57.6 |  | 322.014 |  |
| 58.5 |  | 317.074 |  |
| 58.5 |  | 322.024 |  |
| 59 |  |  | 240.105 |
| 59.1 |  | 240.095 |  |
| 59.3 |  | 317.078 |  |
| 59.3 |  | 322.027 |  |
| 60.1–61.2 |  | 254.17 | 254.172 |
| 60.2 |  |  | 213.144 |
| 60.9 |  | 375.166 |  |
| 60.9–61.3 |  | 392.179 | 392.208 |
| 61.3 |  |  | 208.028 |
| 64.8 |  |  | 255.182 |
| 70 | 348.146 |  |  |
| 71.65 | 348.147 |  |  |
| 78.8 |  |  | 331.021 |
| 83.027–83.2 |  | 325.267 | 325.286 |
| **Total** | 15 | 83 | 32 |
