# Supplementary material for: Methods matter: Comparison of techniques used for sea anemone venom extraction
Source: Toxicon X. 2025 Mar 8;26:100219. doi: 10.1016/j.toxcx.2025.100219 (PMC11954122; doi:10.1016/j.toxcx.2025.100219)
Supplement: Supplementary Table 2 [file mmc2.doc]

**Methods Matter: Comparison of techniques used for sea anemone venom extraction**

Authors: Kaposi K.L^1^,^2^*, Wilson, D.T^2^, Jones, A.^3^ and Seymour J.E^2^

**Supplementary Table 2:** Monoisotopic mass (Da) and retention time (min) of molecules identified from within peaks of interest seen within the 280 nm chromatogram for the >3 kDa venom fractions collected from sea anemone *Isactinia*-MTQ, using the isolated cnidae (Cn), electrostimulation (E), and physical manipulation (Pm) methods.

| **Retention Time (min)** | **Monoisotopic Mass (Da)** | | |
| --- | --- | --- | --- |
|  | **Cn** | **E** | **Pm** |
| 3.6 |  |  | 315.971 |
| 4.5 |  |  | 180.952 |
| 4.5 |  |  | 366.062 |
| 5.2 |  | 1887.06 |  |
| 5.6 |  |  | 510.177 |
| 7.6 |  |  | 164.952 |
| 10.2 |  |  | 1730.55 |
| 10.2 |  |  | 1886.93 |
| 10.8 |  |  | 3783.86 |
| 10.8 |  |  | 3947.45 |
| 11.2 |  |  | 1587.31 |
| 11.2 |  |  | 3931.33 |
| 11.2 |  |  | 4029.11 |
| 12 |  | 2130.93 |  |
| 12 |  | 3930.78 |  |
| 12.3 |  |  | 203.955 |
| 12.3 |  |  | 3687.77 |
| 14.6 |  | 3046.22 |  |
| 14.8 |  |  | 3762.71 |
| 14.8 |  |  | 3860.92 |
| 15.6 |  |  | 3525.61 |
| 15.9–16.1 |  | 5687.88 | 5687.6 |
| 16.7 |  |  | 3744.88 |
| 17.6 |  |  | 2772.13 |
| 17.6 |  |  | 3726.52 |
| 19 |  |  | 4182.48 |
| 19 |  |  | 6737.03 |
| 20.3 |  |  | 4328.86 |
| 20.3 |  |  | 5241.64 |
| 20.8 |  | 4232.43 |  |
| 21.5–21.7 |  | 3923.94 | 3923.75 |
| 21.7 |  | 4242.59 |  |
| 22.8 |  |  | 4686.54 |
| 23.6 |  | 4686.4 |  |
| 25.4–25.5 |  | 4925.44 | 4925.44 |
| 29.4 |  |  | 4855.47 |
| 30.4 | 6714.17 |  |  |
| 34.4 |  |  | 9162.82 |
| 31.1–31.4 |  | 4898.8 | 4898.56 |
| 35 |  | 14608.7 |  |
| 36 | 227.06 |  |  |
| 44.1 | 16250.7 |  |  |
| **Total** | 3 | 12 | 31 |
