# Supplementary material for: Methods matter: Comparison of techniques used for sea anemone venom extraction
Source: Toxicon X. 2025 Mar 8;26:100219. doi: 10.1016/j.toxcx.2025.100219 (PMC11954122; doi:10.1016/j.toxcx.2025.100219)
Supplement: Supplementary Table 3 [file mmc3.doc]

**Methods Matter: Comparison of techniques used for sea anemone venom extraction**

Authors: Kaposi K.L^1^,^2^*, Wilson, D.T^2^, Jones, A.^3^ and Seymour J.E^2^

**Supplementary Table 3:** Monoisotopic mass (Da) and retention time (min) of molecules identified from the total ion content (TIC) spectra obtained via uHPLC-MS analysis of <3kDa venom fractions collected from sea anemone *Isactinia*-MTQ, using the isolated cnidae (Cn), electrostimulation (E), and physical manipulation (Pm) methods. Molecules marked with an * are considered likely to be the same, but exhibit a higher degree of uncertainty due to low resolution and intensity.

| **Retention Time (min)** | **Monoisotopic Mass (Da)** | | |
| --- | --- | --- | --- |
|  | **Cn** | **E** | **Pm** |
| 4.29 | 691.1957 |  |  |
| 4.32 | 769.2904 |  |  |
| 4.34 | 657.2115 |  |  |
| 4.34 | 700.3392 |  |  |
| 4.36 |  | 665.7054 |  |
| 4.37 |  | 567.7394 |  |
| 4.37 |  | 687.6899 |  |
| 4.37 | 786.3446 |  |  |
| 4.39 |  | 545.7518 |  |
| 4.39–5.49 | 524.2438* | 523.7747* |  |
| 4.41 |  | 403.8233 |  |
| 4.41 | 873.2486 |  |  |
| 4.41 | 1016.3528 |  |  |
| 4.41–7.49 | 643.3902* | 643.7254* |  |
| 4.42 |  | 763.6784 |  |
| 4.43 | 742.2373 |  |  |
| 4.43 | 792.2982 |  |  |
| 4.44 | 610.1914 |  |  |
| 4.44 |  | 621.7447 |  |
| 4.44 |  | 741.6964 |  |
| 4.44–4.46 | 719.3105 | 719.7062 |  |
| 4.44–7.52 | 502.2570* | 501.7917* |  |
| 4.45 | 726.2417 |  |  |
| 4.45 | 753.2986 |  |  |
| 4.46 |  | 479.803 |  |
| 4.57 | 644.1498 |  |  |
| 4.62 | 508.2811 |  |  |
| 4.66 | 624.3083 |  |  |
| 4.69 | 424.2056 |  |  |
| 4.69 | 611.1225 |  |  |
| 4.71 | 553.2688 |  |  |
| 5.02 | 486.2729 |  |  |
| 5.55 | 459.2619 |  |  |
| 5.61 | 493.2029 |  |  |
| 5.62 | 582.3184 |  |  |
| 5.63 | 414.2349 |  |  |
| 5.63 | 530.298 |  |  |
| 5.64 | 574.3094 |  |  |
| 5.65 | 588.3187 |  |  |
| 5.65 | 1235.6113 |  |  |
| 5.71 | 435.2353 |  |  |
| 5.71 | 572.3094 |  |  |
| 5.74 | 468.2014 |  |  |
| 5.76 | 424.2118 |  |  |
| 5.76 | 442.2232 |  |  |
| 5.76 | 465.2384 |  |  |
| 5.76 | 471.2207 |  |  |
| 5.78 | 570.2846 |  |  |
| 5.81 | 475.2434 |  |  |
| 5.83 | 444.2351 |  |  |
| 6.01 | 408.1934 |  |  |
| 6.03 | 1110.3044 |  |  |
| 6.04 | 626.1167 |  |  |
| 6.04 | 808.2369 |  |  |
| 6.55 | 573.3432 |  |  |
| 6.56 | 447.2173 |  |  |
| 6.57 | 515.3255 |  |  |
| 6.58 | 566.2568 |  |  |
| 6.59 | 625.2514 |  |  |
| 6.62 | 450.2434 |  |  |
| 6.62 | 594.2892 |  |  |
| 6.64 | 599.2785 |  |  |
| 6.7 | 620.2845 |  |  |
| 6.7 | 653.2935 |  |  |
| 6.75 | 520.2469 |  |  |
| 6.77 | 509.2311 |  |  |
| 6.78 | 474.2497 |  |  |
| 6.78 | 592.2949 |  |  |
| 6.83 | 544.3112 |  |  |
| 6.84 | 570.2824 |  |  |
| 6.87 | 423.1884 |  |  |
| 6.89 | 537.2291 |  |  |
| 7.33 | 461.2287 |  |  |
| 7.34 | 533.2359 |  |  |
| 7.37 | 434.2153 |  |  |
| 7.38 | 480.2113 |  |  |
| 7.47 | 863.4654 |  |  |
| 7.59 | 539.2418 |  |  |
| 7.62 | 772.3996 |  |  |
| 7.99 | 548.2047 |  |  |
| 8.06 | 500.2766 |  |  |
| 8.06 | 665.2848 |  |  |
| 8.14 | 707.2426 |  |  |
| 8.15 | 542.3263 |  |  |
| 8.17 | 459.2477 |  |  |
| 8.21 | 652.2942 |  |  |
| 8.27 | 418.2202 |  |  |
| 8.64 | 502.2949 |  |  |
| 8.72 | 409.1529 |  |  |
| 8.76 | 590.2794 |  |  |
| 8.76 | 956.527 |  |  |
| 8.83 | 638.2842 |  |  |
| 8.89 | 414.2204 |  |  |
| 8.89 | 602.318 |  |  |
| 8.93 | 475.2482 |  |  |
| 8.95 | 430.2209 |  |  |
| 9.29 | 423.1783 |  |  |
| 9.35 | 485.3128 |  |  |
| 9.35 | 558.2681 |  |  |
| 9.42 | 546.2858 |  |  |
| 9.43 | 432.2396 |  |  |
| 9.43 | 697.2823 |  |  |
| 9.44 | 582.3375 |  |  |
| 9.48 | 401.2185 |  |  |
| 9.86 | 731.3704 |  |  |
| 9.86 | 816.4324 |  |  |
| 9.86 | 826.3496 |  |  |
| 9.9 | 729.3763 |  |  |
| 9.9 | 4853.1686 |  |  |
| 9.91 | 609.2523 |  |  |
| 9.94 | 492.2325 |  |  |
| 9.96 | 645.3052 |  |  |
| 10.39 | 457.2295 |  |  |
| 10.41 | 430.2222 |  |  |
| 10.48 | 423.1776 |  |  |
| 10.58 | 589.283 |  |  |
| 10.71 | 507.2789 |  |  |
| 10.75 | 491.2279 |  |  |
| 10.77 | 567.2768 |  |  |
| 10.9 | 428.2459 |  |  |
| 10.94 | 423.2134 |  |  |
| 10.96 | 750.3175 |  |  |
| 10.97 | 677.3143 |  |  |
| 11.03 | 682.2665 |  |  |
| 11.19 | 653.2799 |  |  |
| 11.2 | 587.3054 |  |  |
| 11.27 | 430.2138 |  |  |
| 11.3 | 459.2278 |  |  |
| 11.43 | 409.2005 |  |  |
| 11.44 | 762.4095 |  |  |
| 11.45 | 849.4429 |  |  |
| 11.48 | 471.242 |  |  |
| 11.48 | 784.3897 |  |  |
| 11.67 | 591.2848 |  |  |
| 11.77 | 487.277 |  |  |
| 11.78 | 434.2001 |  |  |
| 11.85 | 901.4373 |  |  |
| 11.86 | 494.2581 |  |  |
| 11.92 | 585.2479 |  |  |
| 11.95 | 696.2837 |  |  |
| 12.13 | 676.3063 |  |  |
| 12.16 | 538.2427 |  |  |
| 12.19 | 674.3039 |  |  |
| 12.21 | 428.2453 |  |  |
| 12.39 | 520.2653 |  |  |
| 12.48 | 505.2375 |  |  |
| 12.56 | 423.2096 |  |  |
| 12.65 | 936.4062 |  |  |
| 12.81 | 506.2127 |  |  |
| 12.9 | 529.2915 |  |  |
| 12.92 | 416.2467 |  |  |
| 13.32 | 625.2848 |  |  |
| 13.51 | 473.2524 |  |  |
| 13.51 | 492.2408 |  |  |
| 13.66 | 575.2968 |  |  |
| 13.69 | 459.2517 |  |  |
| 13.83 | 648.3216 |  |  |
| 13.86 | 467.3015 |  |  |
| 13.93 | 592.2667 |  |  |
| 13.94 | 501.2581 |  |  |
| 13.98 | 667.4293 |  |  |
| 14.01 | 550.2587 |  |  |
| 14.18 | 432.2221 |  |  |
| 14.22 | 430.2392 |  |  |
| 14.26 | 418.2146 |  |  |
| 14.27 | 561.2866 |  |  |
| 14.3 | 1055.5757 |  |  |
| 14.34 | 442.2552 |  |  |
| 14.44 | 473.2677 |  |  |
| 14.5 | 574.3241 |  |  |
| 14.55 | 588.2923 |  |  |
| 14.56 | 584.3398 |  |  |
| 14.58 | 402.2233 |  |  |
| 14.65 | 890.5094 |  |  |
| 14.78 | 416.2348 |  |  |
| 14.83 | 777.3892 |  |  |
| 15.03 | 507.2721 |  |  |
| 15.06–16.06 | 436.1624 |  | 436.245 |
| 15.19 | 474.23 |  |  |
| 15.22 | 416.2435 |  |  |
| 15.25 | 760.427 |  |  |
| 15.27 | 418.2055 |  |  |
| 15.29 | 513.2999 |  |  |
| 15.31 | 549.3091 |  |  |
| 15.36 | 782.459 |  |  |
| 15.4 | 1121.6067 |  |  |
| 15.41 | 725.3513 |  |  |
| 15.48 | 477.2385 |  |  |
| 15.48 | 597.2885 |  |  |
| 15.49 | 831.4664 |  |  |
| 15.58 | 418.2498 |  |  |
| 15.6 | 611.2839 |  |  |
| 15.65 | 679.2563 |  |  |
| 15.68 | 545.2812 |  |  |
| 15.75 | 603.3337 |  |  |
| 15.76 | 1145.6853 |  |  |
| 15.94 | 583.2184 |  |  |
| 15.95 | 414.2628 |  |  |
| 15.96–16.16 | 431.2912 | 431.2885 | 431.289 |
| 16.02 | 684.374 |  |  |
| 16.08 | 445.2698 |  |  |
| 16.17 | 1014.5616 |  |  |
| 16.18 | 473.2641 |  |  |
| 16.19 | 916.6016 |  |  |
| 16.4 | 430.2566 |  |  |
| 16.59 | 989.5717 |  |  |
| 16.69 | 474.2445 |  |  |
| 16.7 | 724.3567 |  |  |
| 17.0 | 632.3246 |  |  |
| 17.01 | 665.3245 |  |  |
| 17.04 | 527.3095 |  |  |
| 17.06–17.13 | 452.3546 | 452.3523 | 452.353 |
| 17.13 |  | 497.4101 | 497.411 |
| 17.23 | 563.3127 |  |  |
| 17.29 | 591.3516 |  |  |
| 17.49–17.57 | 475.3191 | 475.316 | 475.318 |
| 17.51 | 458.2907 |  |  |
| 17.63 | 432.2393 |  |  |
| 17.64 | 489.3001 |  |  |
| 17.66 | 415.2509 |  |  |
| 17.72 | 450.2335 |  |  |
| 17.73 | 735.3695 |  |  |
| 17.81 | 984.5182 |  |  |
| 17.92 | 429.2584 |  |  |
| 18.01 | 407.2258 |  |  |
| 18.01 | 636.3382 |  |  |
| 18.03 | 474.2419 |  |  |
| 18.06 | 629.3514 |  |  |
| 18.14 | 1113.5685 |  |  |
| 18.2 | 487.285 |  |  |
| 18.35 | 633.3595 |  |  |
| 18.38 | 608.3043 |  |  |
| 18.45 | 766.4023 |  |  |
| 18.56 | 508.2367 |  |  |
| 18.84–18.89 | 519.3482 | 519.3433 | 519.345 |
| 18.91 | 502.3196 |  |  |
| 19.02 | 456.2741 |  |  |
| 19.04 | 533.329 |  |  |
| 19.23 | 494.2408 |  |  |
| 19.3 | 1345.6827 |  |  |
| 19.44 | 758.4499 |  |  |
| 19.48 | 1551.8327 |  |  |
| 19.5 | 551.2802 |  |  |
| 19.59 | 805.4011 |  |  |
| 19.62 | 765.353 |  |  |
| 19.64 | 694.3088 |  |  |
| 19.64 | 696.276 |  |  |
| 20.05 | 785.3948 |  |  |
| 20.09–20.11 | 563.3764 | 563.3689 | 563.372 |
| 20.1 | 558.3608 |  |  |
| 20.1 | 561.2984 |  |  |
| 20.12 | 568.3336 |  |  |
| 20.13 | 491.2934 |  |  |
| 20.24 | 458.2948 |  |  |
| 20.26 | 513.3004 |  |  |
| 20.3 | 1258.7518 |  |  |
| 20.4 | 1003.5895 |  |  |
| 20.48–20.5 |  | 565.4369 | 565.44 |
| 20.5 | 513.3096 |  |  |
| 20.5 |  |  | 587.418 |
| 20.5 |  |  | 610.499 |
| 20.5 | 773.3787 |  |  |
| 20.51 | 1501.8676 |  |  |
| 20.58 | 870.5195 |  |  |
| 20.64 | 817.4268 |  |  |
| 20.81 | 484.2146 |  |  |
| 20.81 | 547.284 |  |  |
| 21 | 644.4035 |  |  |
| 21.04 | 688.3837 |  |  |
| 21.23–21.26 | 607.4047 | 607.3955 | 607.399 |
| 21.38 | 1102.6664 |  |  |
| 21.42 | 809.3847 |  |  |
| 21.43 | 787.3978 |  |  |
| 21.44 | 804.423 |  |  |
| 21.48 | 1708.8495 |  |  |
| 21.57 | 434.2741 |  |  |
| 21.64 | 788.3974 |  |  |
| 21.68 | 529.2746 |  |  |
| 21.94 | 453.1778 |  |  |
| 22.18 | 784.419 |  |  |
| 22.19 | 651.4335 |  |  |
| 22.32 | 851.4874 |  |  |
| 22.34 | 1701.9685 |  |  |
| 23.03 | 1428.8199 |  |  |
| 23.16 | 530.2995 |  |  |
| 23.2 | 695.4619 |  |  |
| 23.43 | 452.2853 |  |  |
| 23.51 | 753.4247 |  |  |
| 23.54 | 715.3993 |  |  |
| 23.7 | 679.3192 |  |  |
| 23.71 | 1614.9709 |  |  |
| 24.03 | 739.4885 |  |  |
| 24.08 | 582.3641 |  |  |
| 24.39 | 1005.5452 |  |  |
| 24.51 | 590.3325 |  |  |
| 24.74 | 416.2187 |  |  |
| 24.86 | 411.2658 |  |  |
| 25.02 | 621.3273 |  |  |
| 25.68 | 1686.9655 |  |  |
| 26.12 | 438.2626 |  |  |
| 26.13 | 455.2942 |  |  |
| 26.16 | 552.281 |  |  |
| 28.65 | 415.3124 |  |  |
| 28.92 | 415.3125 |  |  |
| 36.66 | 479.2965 |  |  |
| 37.53 | 408.1725 |  |  |
| 37.54 | 403.2189 |  |  |
| 37.54 | 431.2515 |  |  |
| 37.59 | 789.4115 |  |  |
| 37.97 | 420.3291 |  |  |
| 40.46 | 425.3198 |  |  |
| 41.78 | 414.2251 |  |  |
| 50.99 |  | 485.8375 |  |
| **Total** | 299 | 22 | 11 |
