# Supplementary material for: Methods matter: Comparison of techniques used for sea anemone venom extraction
Source: Toxicon X. 2025 Mar 8;26:100219. doi: 10.1016/j.toxcx.2025.100219 (PMC11954122; doi:10.1016/j.toxcx.2025.100219)
Supplement: Supplementary Table 4 [file mmc4.doc]

**Methods Matter: Comparison of techniques used for sea anemone venom extraction**

Authors: Kaposi K.L^1^,^2^*, Wilson, D.T^2^, Jones, A.^3^ and Seymour J.E^2^

**Supplementary Table 4:** Monoisotopic mass (Da) and retention time (min) of molecules identified from the total ion content (TIC) spectra obtained via uHPLC-MS analysis of >3kDa venom fractions collected from sea anemone *Isactinia*-MTQ, using the isolated cnidae (Cn), electrostimulation (E), and physical manipulation (Pm) methods. Molecules marked with an * are considered likely to be the same, but exhibit a higher degree of uncertainty due to low resolution and intensity.

| **Retention Time (min)** | **Monoisotopic Mass (Da)** | | |
| --- | --- | --- | --- |
|  | **Cn** | **E** | **Pm** |
| 3.04 |  | 510.2539 |  |
| 3.26 |  | 624.2998 |  |
| 3.36 | 424.2096 |  |  |
| 4.36 | 1957.038 |  |  |
| 4.43 | 753.2943 |  |  |
| 4.55 | 508.282 |  |  |
| 4.58 | 565.3027 |  |  |
| 4.72 |  | 498.1726 |  |
| 5.24 |  |  | 430.2304 |
| 5.38–7.57 |  | 559.2736* | 559.2209* |
| 5.65 | 1741.888 |  |  |
| 5.66 | 513.2733 |  |  |
| 5.68 |  |  | 3797.917 |
| 5.68 |  |  | 3895.904 |
| 5.7 | 1137.636 |  |  |
| 5.72 |  |  | 943.0072 |
| 5.72 |  |  | 991.9939 |
| 5.72 |  |  | 992.4969 |
| 5.72 |  |  | 1886.005 |
| 5.72–5.78 |  | 1983.968 | 1983.985 |
| 5.72–5.78 |  | 2081.932 | 2081.961 |
| 5.73 |  |  | 943.5112 |
| 5.73 |  |  | 1920.009 |
| 5.73 |  |  | 2017.977 |
| 5.73 |  |  | 2082.949 |
| 5.73–5.77 |  | 2179.902 | 2179.927 |
| 5.73 |  |  | 2278.912 |
| 5.74 |  |  | 2376.89 |
| 5.76 |  | 1729.882 |  |
| 5.77 |  | 1827.844 |  |
| 5.78 |  | 1885.997 |  |
| 5.78 |  | 2277.872 |  |
| 5.82 | 424.211 |  |  |
| 5.82 | 442.2225 |  |  |
| 5.83 | 2170.137 |  |  |
| 5.83–6.55 |  | 3781.883* | 3781.931* |
| 5.83 |  |  | 3879.887 |
| 5.83 |  |  | 3977.889 |
| 5.83 |  |  | 4173.854 |
| 5.86 |  |  | 4075.855 |
| 5.87 | 2268.087 |  |  |
| 5.92–7.46 |  | 572.3058* | 572.3134* |
| 5.92 |  | 577.2316 |  |
| 5.98 |  | 929.3978 |  |
| 6.2–6.64 |  | 501.2682 | 501.2729 |
| 6.26 |  |  | 903.3341 |
| 6.55 |  | 530.2947 |  |
| 6.58 | 515.3247 |  |  |
| 6.6 | 543.2718 |  |  |
| 6.62 |  | 472.2877 |  |
| 6.64 |  |  | 4075.872 |
| 6.66 |  |  | 3977.923 |
| 6.69 |  |  | 631.3526 |
| 6.69 | 653.2927 |  |  |
| 6.7 |  | 786.4334 |  |
| 6.71 |  | 488.3428 |  |
| 6.73 | 2302.081 |  |  |
| 6.76 |  | 729.3146 |  |
| 6.77 | 2204.131 |  |  |
| 6.78 |  | 631.3467 |  |
| 6.78 |  | 1294.627 |  |
| 6.8 |  | 1197.682 |  |
| 6.8 |  | 1392.598 |  |
| 6.86 | 537.2297 |  |  |
| 7.45 |  | 1302.669 |  |
| 7.46 |  | 523.2888 |  |
| 7.48 |  | 403.2172 |  |
| 7.48 |  | 953.5154 |  |
| 7.5 |  | 5150.772 |  |
| 7.51 |  | 3617.277 |  |
| 7.54 |  |  | 4042.997 |
| 7.55 |  |  | 3945.027 |
| 7.57 |  | 1059.533 |  |
| 7.57 |  | 1146.576 |  |
| 7.59 |  | 1040.544 |  |
| 7.59 |  | 1957.997 |  |
| 7.64 |  |  | 2153.971 |
| 7.66 |  |  | 2228.045 |
| 7.74 |  |  | 2423.995 |
| 8.07 | 745.3898 |  |  |
| 8.15 |  | 2130.06 |  |
| 8.17 | 1073.502 |  |  |
| 8.19 |  | 2228.029 | 2228.037 |
| 8.2 |  |  | 2423.988 |
| 8.21 |  | 1018.506 |  |
| 8.22 |  |  | 2229.053 |
| 8.24 |  |  | 4027.011 |
| 8.27 |  |  | 561.2955 |
| 8.28 |  | 587.3468 |  |
| 8.78 |  | 610.2803 |  |
| 8.86 |  |  | 3930.032 |
| 8.89 |  |  | 4223.017 |
| 8.91 |  |  | 4026.988 |
| 8.98 |  |  | 801.4565 |
| 9.06 |  | 438.2248 |  |
| 9.08 |  |  | 4027.028 |
| 9.3 |  | 3929.999 |  |
| 9.35 |  | 3928.992 |  |
| 9.37 |  | 614.3098 |  |
| 9.41 | 558.2673 |  |  |
| 9.92 | 728.377 |  |  |
| 9.98 |  |  | 523.2116 |
| 10.05–10.09 |  | 472.2826 | 472.2859 |
| 10.07 |  |  | 947.4635 |
| 10.34 | 515.2637 |  |  |
| 10.41 |  |  | 409.2008 |
| 10.61 | 589.2842 |  |  |
| 10.81 |  |  | 409.2008 |
| 10.89 |  | 592.2684 |  |
| 10.89–10.9 |  | 679.3058 | 679.31 |
| 10.97 |  |  | 409.2003 |
| 10.97 |  | 1170.613 |  |
| 10.98 | 682.2725 |  |  |
| 11 |  | 610.2807 |  |
| 11.02 |  | 1078.591 |  |
| 11.03–11.24 | 660.2895 |  | 660.2904 |
| 11.03 | 706.3536 |  |  |
| 11.04 | 677.3139 |  |  |
| 11.04 |  |  | 833.417 |
| 11.23 |  |  | 705.3493 |
| 11.32–13.24 | 722.388* | 722.3171* |  |
| 11.38 |  | 5394.034 |  |
| 11.46 |  | 553.2575 |  |
| 11.54 | 762.4098 |  |  |
| 11.81 | 1106.546 |  |  |
| 11.93 | 696.2891 |  |  |
| 11.93–12.16 | 719.3643 |  | 719.3637 |
| 11.94 | 691.3291 |  |  |
| 11.96–12.14 | 674.3066 | 674.297 |  |
| 12.14 |  |  | 674.307 |
| 12.17 |  |  | 1443.869 |
| 12.33 |  |  | 2558.213 |
| 12.33 |  |  | 2629.251 |
| 12.41 |  | 551.2778 |  |
| 12.57 |  | 549.3116 |  |
| 12.58 |  | 3044.654 |  |
| 12.58–12.65 |  | 3142.63 | 3142.654 |
| 12.59 |  | 894.4066 |  |
| 12.62 |  |  | 644.3797 |
| 12.64 |  | 1198.686 |  |
| 12.65 |  | 613.2321 |  |
| 12.65 |  |  | 3422.577 |
| 12.81 |  | 3044.65 |  |
| 12.85 |  | 570.3574 |  |
| 12.98 |  | 663.3121 |  |
| 12.99 |  | 606.335 |  |
| 12.99 |  | 778.3821 |  |
| 13.04 |  | 441.2514 |  |
| 13.05 |  |  | 2844.37 |
| 13.31 |  | 592.2716 |  |
| 13.34 |  |  | 631.3485 |
| 13.36 |  | 530.3238 |  |
| 13.37 |  |  | 2771.326 |
| 13.49 |  |  | 2771.351 |
| 13.54 |  |  | 2869.327 |
| 13.55–13.62 |  | 503.3093 | 503.3203 |
| 13.6 | 827.4776 |  |  |
| 13.83 |  |  | 416.2393 |
| 13.83 | 648.3246 |  |  |
| 14.01 |  | 1005.485 |  |
| 14.08 |  | 570.3595 |  |
| 14.2 |  | 778.3821 |  |
| 14.24 |  | 663.315 |  |
| 14.84 | 1294.623 |  |  |
| 14.85 | 2655.326 |  |  |
| 14.96 |  | 3823.064 |  |
| 15.03 |  | 3725.085 |  |
| 15.04 |  | 1280.702 |  |
| 15.09 |  | 587.3488 |  |
| 15.32 | 2117.166 |  |  |
| 15.36 | 1121.635 |  |  |
| 15.38–16.25 |  | 490.2602* | 490.2516* |
| 15.5 | 1121.621 |  |  |
| 15.55 | 1115.636 |  |  |
| 15.66 |  | 721.4112 |  |
| 15.66–1569 |  | 4180.261 | 4180.274 |
| 15.67 |  |  | 899.411 |
| 15.7 |  |  | 1701.929 |
| 15.72–15.77 | 1145.686 |  | 1145.686 |
| 15.73 | 761.3631 |  |  |
| 15.73 |  | 821.3924 |  |
| 15.73 |  |  | 3185.579 |
| 15.79–15.91 |  | 3142.567 | 3142.567 |
| 15.89 |  | 1994.943 |  |
| 15.92 |  |  | 3185.563 |
| 15.93–15.94 |  | 4180.26 | 4180.081 |
| 15.94 |  |  | 3209.464 |
| 15.96 |  |  | 4180.339 |
| 15.97 |  |  | 4278.378 |
| 15.98 |  |  | 540.318 |
| 15.99 | 431.2921 |  |  |
| 15.99 |  | 8656.831 |  |
| 16.1–16.18 |  | 3112.545 | 3112.585 |
| 16.11 | 1454.997 |  |  |
| 16.16 |  |  | 3210.536 |
| 16.18 |  | 3098.524 |  |
| 16.32 |  | 580.2535 |  |
| 16.33 | 1310.669 |  |  |
| 16.39 |  | 565.2693 |  |
| 16.41 | 916.6027 |  |  |
| 16.52 |  | 536.3024 |  |
| 16.62 |  | 821.372 |  |
| 16.79 | 989.5745 |  |  |
| 16.83–17.33 |  | 452.3567 | 452.3575 |
| 16.88 |  | 3813.66 |  |
| 17.07 |  | 1542.746 |  |
| 17.11 | 1219.719 |  |  |
| 17.19 |  | 565.2627 |  |
| 17.41 | 903.5031 |  |  |
| 17.45 |  | 623.3519 |  |
| 17.5–17.65 |  | 475.3152 | 475.312 |
| 17.59 | 1380.691 |  |  |
| 17.62 |  |  | 696.455 |
| 17.72 |  | 2602.191 |  |
| 17.89 | 735.3826 |  |  |
| 18.00 |  | 4230.461 |  |
| 18.01 | 628.358 |  |  |
| 18.11 |  | 677.4226 |  |
| 18.22 |  | 546.322 |  |
| 18.23 | 2319.216 |  |  |
| 18.29 |  |  | 475.2566 |
| 18.3 |  |  | 3922.151 |
| 18.3 |  |  | 4020.074 |
| 18.31 | 1576.932 |  |  |
| 18.35 |  | 412.2875 |  |
| 18.51 |  | 450.2673 |  |
| 18.66 |  | 450.2717 |  |
| 18.68 |  | 1345.78 |  |
| 18.68 | 4629.888 |  |  |
| 18.73 | 2071.213 |  |  |
| 18.77 |  | 1057.628 |  |
| 18.78 | 1670.918 |  |  |
| 18.87 |  | 1232.778 |  |
| 18.9 | 519.3519 |  |  |
| 18.9 |  |  | 1961.503 |
| 18.92 |  |  | 4310.461 |
| 19.04 |  | 531.3546 |  |
| 19.13–21.59 | 787.4043 | 787.4886 | 787.4079 |
| 19.33 | 1265.663 |  |  |
| 19.39 |  |  | 4312.259 |
| 19.43 | 1020.552 |  |  |
| 19.5 | 1551.836 |  |  |
| 19.56 | 1562.882 |  |  |
| 19.62 |  | 2815.583 |  |
| 19.7 | 1595.946 |  |  |
| 19.76 | 835.4863 |  |  |
| 19.86 | 836.4964 |  |  |
| 19.89 | 1411.807 |  |  |
| 19.92 |  | 1425.872 |  |
| 19.99 | 1359.709 |  |  |
| 20.05 |  | 3098.776 |  |
| 20.08 |  | 3472.973 |  |
| 20.11 |  | 551.3223 |  |
| 20.14 |  | 1297.606 |  |
| 20.16–20.32 | 563.3819 |  | 563.3807 |
| 20.16 |  | 3543.999 |  |
| 20.25 |  | 558.3665 |  |
| 20.27 |  | 1681.855 |  |
| 20.37 |  | 2588.139 |  |
| 20.38 | 1612.006 |  |  |
| 20.5–20.68 | 773.3901 | 773.3928 | 773.3892 |
| 20.53 | 1501.882 |  |  |
| 20.66 |  |  | 565.4498 |
| 20.79 |  |  | 975.5151 |
| 20.86 |  | 2442.415 |  |
| 20.87 |  | 1804.698 |  |
| 20.89 | 2143.145 |  |  |
| 20.9 |  |  | 975.5135 |
| 20.94 |  | 2344.458 |  |
| 21.03 |  |  | 5021.26 |
| 21.11–21.28 |  | 487.3437 | 487.3359 |
| 21.17 |  |  | 5021.196 |
| 21.21 | 2125.161 |  |  |
| 21.24 |  |  | 4928.113 |
| 21.34 |  | 607.4091 |  |
| 21.38 |  | 664.3813 |  |
| 21.46 | 1600.046 |  |  |
| 21.47 |  |  | 487.358 |
| 21.58 |  |  | 4603.42 |
| 21.65 | 1708.878 |  |  |
| 21.65 |  | 1789.975 |  |
| 21.67 | 1102.68 |  |  |
| 21.68 | 1880.164 |  |  |
| 21.7 |  | 2659.535 |  |
| 21.71 |  | 1844.936 |  |
| 21.79 | 1893.139 |  |  |
| 21.86 | 3486.763 |  |  |
| 21.87 |  | 1844.885 |  |
| 21.89 |  | 1356.691 |  |
| 21.89 |  | 1356.696 |  |
| 21.93 |  | 1717.944 |  |
| 21.95 |  |  | 4684.573 |
| 21.96 |  | 1718.952 |  |
| 22.01 |  | 1899.784 |  |
| 22.04 |  |  | 3066.418 |
| 22.05 |  | 1356.674 |  |
| 22.13 |  | 1282.711 |  |
| 22.14 |  |  | 4505.562 |
| 22.23 | 2112.165 |  |  |
| 22.23 |  |  | 4686.407 |
| 22.26 |  |  | 3066.36 |
| 22.27 |  |  | 4509.349 |
| 22.28 |  |  | 4506.486 |
| 22.4 | 1701.994 |  |  |
| 22.48 |  | 1917.798 |  |
| 22.49 |  | 1169.603 |  |
| 22.55 |  | 1168.592 |  |
| 22.62 |  | 926.4649 |  |
| 22.64 | 2211.416 |  |  |
| 22.66 |  | 1538.807 |  |
| 22.66 |  | 3691.087 |  |
| 22.72 | 3031.775 |  |  |
| 22.78 |  | 926.4734 |  |
| 22.81 |  | 3065.363 |  |
| 22.96 |  | 1609.866 |  |
| 23.00 |  |  | 3065.379 |
| 23.16 |  |  | 1183.588 |
| 23.17 | 1693.936 |  |  |
| 23.42 |  | 1356.835 |  |
| 23.48 |  | 1700.916 |  |
| 23.56 |  | 2806.631 |  |
| 23.59 | 1895.119 |  |  |
| 23.62 | 1614.992 |  |  |
| 23.62 |  |  | 4782.455 |
| 23.68 |  |  | 4684.836 |
| 23.72 |  | 1018.61 |  |
| 23.78 |  |  | 4686.333 |
| 23.84 | 1724.024 |  |  |
| 24.08 |  | 1056.692 |  |
| 24.15 |  |  | 4869.649 |
| 24.22 | 582.3711 |  |  |
| 24.49 |  |  | 4058.857 |
| 24.69 |  |  | 675.3986 |
| 24.86 |  |  | 4869.489 |
| 24.9 |  | 685.4399 |  |
| 24.96 |  |  | 4852.421 |
| 25.01 |  | 4546.452 |  |
| 25.13 |  |  | 4853.583 |
| 25.24 | 1745.98 |  |  |
| 25.27 |  |  | 4853.68 |
| 25.37 | 2178.425 |  |  |
| 25.42 |  |  | 675.4027 |
| 25.48 |  | 676.4031 |  |
| 25.56 |  | 588.3599 |  |
| 25.56 |  |  | 4868.667 |
| 25.57 | 1687.983 |  |  |
| 25.59 |  | 676.4139 |  |
| 25.76 |  |  | 4968.395 |
| 25.77 |  |  | 4852.652 |
| 25.82 | 1686.969 |  |  |
| 26.01 | 12314.63 |  |  |
| 26.18 |  |  | 4951.586 |
| 26.33 |  |  | 4952.62 |
| 26.45 |  |  | 4950.7 |
| 26.48 | 2225.256 |  |  |
| 26.48 |  |  | 4954.659 |
| 26.48 |  |  | 7282.119 |
| 27.16 |  | 757.4493 |  |
| 27.22 |  | 1938.959 |  |
| 27.67 |  |  | 4994.728 |
| 28.07 |  |  | 4994.699 |
| 28.38 | 2804.903 |  |  |
| 28.38 |  |  | 4897.646 |
| 28.41 |  |  | 4994.739 |
| 28.72 |  | 3165.725 |  |
| 28.81 | 2735.537 |  |  |
| 28.84 |  | 3165.738 |  |
| 28.85 |  | 1469.946 |  |
| 28.94 | 9771.806 |  |  |
| 29.11 | 2722.544 |  |  |
| 29.11 |  | 14608.01 |  |
| 29.16 |  | 14710.42 |  |
| 29.24 |  |  | 5306.827 |
| 29.29 |  | 789.4436 |  |
| 29.30 |  | 3652.404 |  |
| 29.59 |  | 892.5294 |  |
| 29.65 |  |  | 5307.849 |
| 29.95 |  | 9166.405 |  |
| 30.00 |  | 9164.468 | 9165.196 |
| 30.14 |  |  | 1326.957 |
| 30.17 |  |  | 5405.88 |
| 30.17 |  |  | 5406.911 |
| 30.24 | 8595.769 |  |  |
| 30.26 | 4205.067 |  |  |
| 38.19 |  | 420.3417 |  |
| 40.00 |  |  | 597.3731 |
| 41.52 | 463.3053 |  |  |
| 42.25 | 481.317 |  |  |
| 46.18 |  |  | 499.3058 |
| 48.51–48.57 | 501.3226 | 501.3229 | 501.3212 |
| 48.56 |  |  | 1002.653 |
| 48.68 |  |  | 523.3045 |
| 49.25–49.31 | 479.3723 |  | 479.3667 |
| 49.33 | 465.3187 |  |  |
| 49.34–49.35 |  | 465.318 | 465.3153 |
| 49.41 |  |  | 930.6414 |
| 49.74 |  |  | 465.3108 |
| 49.92 | 531.4113 |  |  |
| 50.1–50.16 | 575.441 | 575.4427 |  |
| 50.19 | 663.5007 |  |  |
| 50.2 | 619.4714 |  |  |
| 50.25 |  | 619.4731 |  |
| 50.29 | 707.5309 |  |  |
| 50.31 | 451.3009 |  |  |
| 50.36 | 751.5597 |  |  |
| 50.36 | 795.586 |  |  |
| 50.39 | 467.3695 |  |  |
| 51.3–51.47 | 495.3677 |  | 495.3652 |
| 51.6 |  |  | 481.3504 |
| **Total** | 119 | 174 | 153 |
